# Why Should Medical Students Care about Health Policy?

**DOI:** 10.1371/journal.pmed.0030199

**Published:** 2006-10-24

**Authors:** Rajesh Gupta

## Abstract

Gupta argues that medicine has become increasingly influenced by political, economic and social policies, and that medical students should become well-versed in these policies.

As the practice of medicine becomes increasingly influenced by political, economic, and social policies, it is crucial that medical students become well versed in this expanded vision of “health policy.” However, the current medical curriculum at nearly all medical schools contains very little formal education and training in this area. In this Student Forum, I argue for improvement in medical education, to cultivate future “physician/policy scientists.”

## Redefining Health Policy and the Practice of Medicine

Traditionally, the field of “health policy” has referred to medical policies affecting the health of people. However, many have argued that political, social, and economic policies have an equal, and sometimes greater, influence on the health of populations [1–4]. For example, economic sanctions in Iraq and Cuba were intended as political punishment for those in power, but ultimately they led to increased infant morbidity and mortality, respectively [5,6]. Genocide campaigns, coupled with their direct effects on morbidity and mortality, tend to result in the formation of refugee camps plagued with disease [7,8]. In addition, international trade agreements affect the availability of key drugs for the treatment of many communicable and noncommunicable diseases [9,10]. Pharmaceutical companies have, thus far, mostly focused their efforts on the needs of the wealthy minority rather than diseases affecting the poor majority [11], although there are encouraging signs that drug companies are now becoming interested in neglected diseases of poverty [12]. Financial counsel and policies from institutions such as the World Bank and the International Monetary Fund to resource-poor countries, unintentionally, have been linked to increases in disease morbidity [1]. Health-related benefits from technological advances may be limited if the underlying determinants of health are not equally addressed [13]. Political, economic, and social policies have direct downstream consequences on health, and, unfortunately, it is the poor that suffer the most from this “systemic dysfunction [with]in [our] complex world” [14].

Political, economic, and social policies affect health through their direct effects on both prevention and treatment policies—the two interlinked foundations of modern medicine. For example, epidemiological risk factors are important in reference to prevention efforts, but we must also examine the core issues that give rise to those risk factors (i.e., the biosocial determinants of health). The nonmedical determinants of health can influence the behavior of populations to the point of pushing selective groups into “high-risk” categories. Treatment is affected by both clinical knowledge and the diagnostic/therapeutic tools available to the physician. In turn, the effectiveness of these tools is often influenced by policies affecting issues such as access to clean water and electricity. Thus, the field of health policy cannot be limited to only medical policies, but must also include these equally influential, nonmedical determinants of health.

## Women's Health in Haiti

A brief examination of health in Haiti further highlights how health policies must incorporate the nonmedical determinants of health. In Haiti (and in other countries), commercial sex workers (CSWs) are at risk for sexually transmitted infections (including HIV). This epidemiological association is built upon a socioeconomic principle that many CSWs do not “choose” to become CSWs but rather become CSWs out of necessity for survival. Once commercial farmers, these individuals were displaced from their land ultimately as a result of political and economic policies, with no option of an alternative source of income. Structural adjustment programs are a good example of a policy that has led to displacement—these programs involve economic policies such as currency devaluation and trade liberalization, which countries must follow in order to qualify for World Bank and International Monetary Fund loans. Thus, as a result of the political, economic, and social history of Haiti, sexual relations as a way of economic survival remains the only choice for many women in the country [15].

The role of the physician needs to be expanded to include the notion of the policy scientist.

An effective strategy to address HIV in Haiti will require a synergistic combination of prevention and treatment. Prevention efforts should not only focus on condom provision but also target those political and economic policies placing populations at risk for becoming CSWs. As a cross-cultural example, an adolescent “street girl” in Rwanda, working as a CSW, stated the following in one interview: “Of course I know we should use condoms. But let me tell you this. I get paid the equivalent of US 25¢ a night if I ask the man to use a condom and 50¢ a night if we do not use one. My family is starving at home. What would you do?” (J. Furin, personal communication).

For those CSWs already infected with HIV and progressing to AIDS, treatment with antiretrovirals (ARVs) has only recently become a viable option. However, the Trade-Related Aspects of Intellectual Property Rights agreement (an international trade law adopted by the World Trade Organization in 1994 that outlines rules and regulations—such as patents and copyrights—for protecting intellectual property) may significantly affect Haiti's (and other poor countries') access to many high-quality, affordable, generic ARVs, as producers in other countries may have to cease production and exportation (http://www.wto.org/english/tratop_e/trips_e/trips_e.htm). Lack of access to ARVs will limit the ways physicians can treat their patients. Thus, prevention and treatment can only be optimal if policies are geared toward truly empowering individuals to use condoms, and if access to ARVs is not impaired or limited.

## Redefining Medical School Training and the Role of the Physician

Ultimately, physicians face a conflict between medical knowledge and the practice of medicine. To address this conflict, and to truly place the needs of patients first, physicians need to be able to put into practice their best understanding of prevention and treatment. This inherently implies being involved in the political, economic, social, and medical affairs affecting the field of health policy.

However, as medical trainees, we focus primarily on clinical knowledge. Once we gain this knowledge, we are then thrust into the practice of medicine. Some individuals ultimately shift toward health policy–oriented careers, with most of that subset focusing on medical policies. What is clearly lacking in this process is the focus on health policy as a career from the outset. Thus, the role of the physician needs to be expanded to include the notion of the policy scientist.

The idea of redefining the role of the physician is not novel, and is exemplified by the concepts of the physician/research scientist and the physician/public health specialist. Such individuals gain training and experience early in their medical school curriculum via joint-degree programs and integration of these disciplines into core class work. Significant training and job opportunities (government, public-sector, and private-sector) are provided, and within many programs exist to foster clinical research or public health as careers. Residency programs, although still extremely oriented toward clinical practice, include tracks designed for clinical research and public health (http://www.amsa.org/global/ih/resprograms.cfm; http://rwjcsp.stanford.edu) [16].

What is needed now is the physician/ policy scientist. Medical schools should structure their curriculum to expose all students to the various aspects of health policy. This would include not only focusing on the medical decision-making process but also on examining how political, economic, and social policies influence health. Formally, the standard medical curriculum should include a specific course devoted to this issue. Schools should offer seminars and training opportunities to further encourage students interested in health policy as a career. Formalized joint-degree programs and internship opportunities at political, economic, social, and medical policy institutions should be developed. Residency programs could be designed to combine clinical training with health policy studies. Finally, specific careers in health policy should be supported by governments, the public sector, and the private sector. Emphasizing health policy from the beginning of the medical education process will properly equip trainees with the skills to be effective in the health policy arena.

## Conclusion

As trainees, we can work hard by narrowly focusing on clinical training. As many of us know and are experiencing, shaping and defining our clinical knowledge is a formidable task that sometimes appears insurmountable. But while clinical knowledge may have been weighted as the dictating force in the practice of medicine in the past, it is no longer the dictating force, nor will it be the force of the future. Political, economic, social, and medical policies of transnational agencies, governments, and the private sector are equal (and sometimes greater) guiding forces in the practice of medicine. We must involve ourselves in these determinants of health as early as possible. And to properly do so, we must support the early development of physicians who delve deeply into these issues.

Trainees should care about health policy because we have chosen a career that requires us to serve our patients, and we have committed ourselves to providing the best standard of care to them. The integrity and the future of our profession now depend upon our influence in the expanding world of health policy, and not solely upon on our clinical knowledge. We will be judged by our actions during this critical stage of health in the world. If we do not act now, we may indeed fail our patients in the future.

## 

**Figure:**
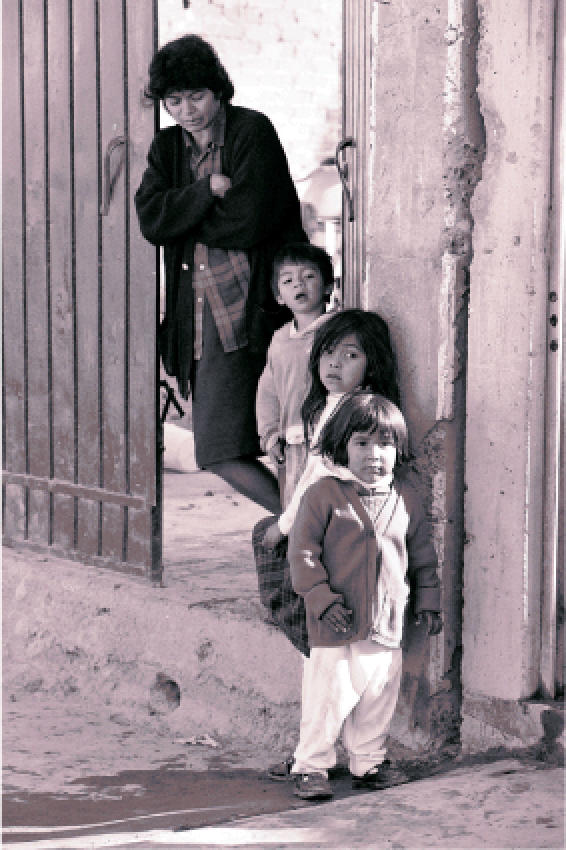
In many countries, the health of women and children is linked with their economic and social status (Photo: Rick Maiman, on behalf of the David and Lucile Packard Foundation)

## Abbreviations

ARV: antiretroviral
CSW: commercial sex worker
